# Review of Vesicular Stomatitis in the United States with Focus on 2019 and 2020 Outbreaks

**DOI:** 10.3390/pathogens10080993

**Published:** 2021-08-06

**Authors:** Angela Pelzel-McCluskey, Brad Christensen, John Humphreys, Miranda Bertram, Robert Keener, Robert Ewing, Lee W. Cohnstaedt, Rachel Tell, Debra P. C. Peters, Luis Rodriguez

**Affiliations:** 1 United States Department of Agriculture (USDA), Animal and Plant Health Inspection Service (APHIS), Veterinary Services, Fort Collins, CO 80526, USA; 2 USDA-APHIS-Veterinary Services, Topeka, KS 66615, USA; Bradley.K.Christensen@usda.gov; 3 USDA-Agriculture Research Service (ARS), Northern Plains Agricultural Research Laboratory, Pest Management Research, Sidney, MT 59270, USA; John.Humphreys@usda.gov; 4 USDA-ARS, Plum Island Animal Disease Center, Long Island, NY 11957, USA; Miranda.Bertram@usda.gov (M.B.); Luis.Rodriguez@usda.gov (L.R.); 5 Department of Agriculture, Fort Hays State University, Hays, KS 67601, USA; rjkeener@fhsu.edu; 6 USDA-ARS, Center for Grain and Animal Health Research, Arthropod-Borne Animal Diseases Research, Manhattan, KS 66502, USA; Robert.Ewing@usda.gov (R.E.); Lee.Cohnstaedt@usda.gov (L.W.C.); 7 USDA-APHIS-Veterinary Services, National Veterinary Services Laboratories, Ames, IA 50010, USA; Rachel.M.Tell@usda.gov; 8 USDA-ARS, Jornada Experimental Range Unit, Las Cruces, NM 88003, USA; Deb.Peters@usda.gov; 9 USDA-ARS SCINet Big Data Program, Berwyn Heights, MD 20740, USA

**Keywords:** vesicular stomatitis, livestock disease, vector-borne disease outbreak, equine

## Abstract

Vesicular stomatitis (VS) is a vector-borne livestock disease caused by vesicular stomatitis New Jersey virus (VSNJV) or vesicular stomatitis Indiana virus (VSIV). The disease circulates endemically in northern South America, Central America, and Mexico and only occasionally causes outbreaks in the United States. Over the past 20 years, VSNJV outbreaks in the southwestern and Rocky Mountain regions occurred with incursion years followed by virus overwintering and subsequent expansion outbreak years. Regulatory response by animal health officials is deployed to prevent spread from lesioned animals. The 2019 VS incursion was the largest in 40 years, lasting from June to December 2019 with 1144 VS-affected premises in 111 counties in eight states (Colorado, Kansas, Nebraska, New Mexico, Oklahoma, Texas, Utah, and Wyoming) and was VSIV serotype, last isolated in 1998. A subsequent expansion occurred from April to October 2020 with 326 VS-affected premises in 70 counties in eight states (Arizona, Arkansas, Kansas, Missouri, Nebraska, New Mexico, Oklahoma, and Texas). The primary serotype in 2020 was VSIV, but a separate incursion of VSNJV occurred in south Texas. Summary characteristics of the outbreaks are presented along with VSV-vector sampling results and phylogenetic analysis of VSIV isolates providing evidence of virus overwintering.

## 1. Introduction

Vesicular stomatitis (VS) is a viral, vector-borne disease of livestock caused by *Vesiculoviruses*, vesicular stomatitis New Jersey virus (VSNJV) or vesicular stomatitis Indiana virus (VSIV), referred to collectively as vesicular stomatitis viruses (VSV). The disease is confined to the Americas where it occurs annually in endemic cycles in Mexico, Central America, and northern regions of South America and only in sporadic epizootic outbreaks every 2–10 years in the United States [1]. Equids, such as horses, mules, and donkeys, are the most commonly affected species in U.S. outbreaks, followed by cattle and camelids, such as llamas and alpacas [2]; however, the disease can also occur in other ruminants and swine. Clinical signs of the disease in affected species are produced by the development of vesicular (blister-like) lesions that occur on the muzzle, nostrils, lips, oral mucosa, tongue, teats, udder, sheath, ventral abdomen, ears, and/or coronary bands [3]. Lesions in the mouth and on the tongue usually cause hypersalivation and anorexia while coronary band lesions often produce lameness. The disease is self-limiting and the lesions in most affected livestock heal within a couple of weeks without veterinary intervention; however, some older animals or those with underlying health conditions may require supportive care, especially in cases with severe oral lesions where the animals cease to eat or drink [3]. The disease is also zoonotic, transmitted to humans through direct contact with infectious lesions in livestock, and typically causes fever, headache, fatigue, and myalgia lasting 3–5 days [3]. The appearance of VSV-caused lesions in ruminants and swine is clinically indistinguishable from lesions of foot and mouth disease (FMD), one of the most economically devastating viral diseases of livestock [1]; therefore, immediate reporting to state and federal animal health officials of VSV-like lesions is required in the U.S. to first rule out FMD infection using appropriate diagnostic assays.

Transmission of VSV to livestock occurs mainly through biting insects [4]; however, spread can also occur through direct contact with virus-containing fluids from infectious lesions and saliva or through indirect contact with contaminated fomites, such as shared water, feed, feeders, lick tubs, tack, or veterinary supplies, such as oral drenching equipment or dental floats [5,6]. Suspected vectors of VSV include black flies (Simuliidae), sand flies (Psychodidae), and *Culicoides* biting midges (Ceratopogonidae) as species from all three of these families have been found to be naturally infected with VSV in the wild [7,8,9]. However, other biting insects have been experimentally infected with VSV and may also be involved in transmission. Proximity of affected livestock premises to water has been indicated as a significant risk factor, which is likely reflective of nearness to prime habitat for competent vectors [10]. Black flies hatching from moving waterways and *Culicoides* spp. hatching from muddy areas around standing water move directly to nearby livestock to feed, thus initiating VSV-transmission in the area if those vector populations are carrying the virus.

Genetic analyses of vesicular stomatitis viruses from U.S. outbreaks have indicated that they arise from viruses circulating in Mexico [11,12,13]. Both VSV serotypes and multiple lineages are found circulating in southern and south-central Mexico annually [1,13,14]. It is hypothesized that specific climatic and environmental factors occur in certain years, which favor expansion of VSV-carrying vectors northward from these endemic regions. In those years, VS cases are seen in states in northern Mexico just a few months prior to outbreaks being recognized in Texas, New Mexico, and/or Arizona in the U.S. These years have been termed incursion years for U.S. outbreaks and the dominant climatological and ecological variables supporting this movement have been modeled and reported [15]. After an incursion year, the virus may overwinter and resurge to cause cases the following year, termed an expansion year, with slightly different climatological and ecological conditions identified as supporting this resurgence [15]. If no VS outbreak is identified in the year following an incursion year, then it is hypothesized that the environmental variables supporting the vectors for an expansion year may not have been present and thus continued transmission did not occur. Research is ongoing to further evaluate and understand how climate and ecology affect insect vector populations and the potential for VSV transmission in a given year.

VS outbreaks in the U.S. cause significant trade disruptions and economic impacts mainly through cessation of international and interstate movement of livestock, but also through reduced participation in or cancellation of livestock shows and events [16]. The seasonality of disease occurrence also has an impact. VS outbreaks occur during the height of vector activity, usually late spring through early fall, which is also the time of year where a high volume of equine shows/events and county fairs are scheduled to occur. Additionally, the large numbers of cattle in the western U.S. that move through livestock markets and sales in the fall can be held up by VS outbreaks and associated quarantines. States without VS cases issue specific movement restrictions on susceptible livestock species from VS-affected states which may bar movement from affected counties altogether or require a certificate of veterinary inspection within just a few days of movement that includes statements by the veterinarian attesting to examination of the animal and the absence of VS lesions. International export of livestock from VS-affected states is halted until at least 30 days after the last quarantine release in the state or longer depending on the requirements of the receiving country. International export of livestock from non-affected U.S. states is usually able to proceed; however, testing for VSV may be required by the receiving country, which adds additional planning and expense to the exporters. While the World Organization for Animal Health (OIE) removed VS from its list of internationally reportable diseases in 2015, the U.S. remains bound by bilateral trade agreements with its trade partners to immediately report the occurrence of VS and provide information on response measures and updates on the outbreak.

## 2. Results

Over the past 20 years, VS outbreaks in the U.S. have been geographically confined mainly to the southwestern and Rocky Mountain regions of the country, have primarily involved the VSNJV serotype of the virus, and large multi-year outbreaks have been temporally separated in 4–8 year increments with smaller, single incursion outbreak years occurring sporadically in between. A summary of outbreak years, affected states, virus serotype, and number of affected livestock premises during this time period is presented in Table 1.

### 2.1. Regulatory Response to VS

Ongoing surveillance for FMD and other foreign vesicular diseases of concern in the U.S. requires that USDA-accredited private veterinarians immediately report to state and federal animal health officials on suspected vesicular lesion occurrence in all livestock species. Follow-up on each report is conducted by a local state or federal veterinary medical officer specifically trained as a foreign animal disease diagnostician (FADD) who deploys to the affected livestock premises, examines the animals, collects the appropriate diagnostic samples, and places a quarantine on the premises. Diagnostic samples are shipped overnight to the USDA’s National Veterinary Services Laboratories (NVSL) in either Ames, Iowa, or Plum Island, New York, depending on the species affected. Samples from vesicular lesioned equids, which cannot be affected with FMD, go to NVSL in Ames, Iowa, with diagnostic testing for VS as the primary rule out, while samples from lesioned ruminants and swine go to NVSL on Plum Island for primary rule out of FMD and foreign swine vesicular diseases, followed by secondary rule out of VS, and tertiary testing for domestic vesicular diseases, such as bluetongue, epizootic hemorrhagic disease, and bovine papular stomatitis in cattle and Senecavirus A in swine.

Diagnostic assays at NVSL used to confirm VSV-infection are specific to each VSV serotype and include antibody detection methods, such as competitive enzyme-linked immunosorbent assay (cELISA), complement fixation test (CFT), virus neutralization (VN), and virus detection methods, such as real-time reverse transcription polymerase chain reaction (rRT-PCR) and virus isolation (VI). While the cELISA is an early indicator of recent infection and will test positive a few days prior to the CFT in a naïve, recently exposed animal, the cELISA may subsequently remain positive for up to 10–12 years [17]. Given the number of previously exposed livestock residing in historically affected regions in the U.S., the cELISA alone cannot be used to confirm recent infection unless occurring in an animal that was either not geographically present in a previous outbreak region or in an animal too young to have experienced the last U.S. outbreak. The CFT, rRT-PCR, and/or VI are used as reliable indicators of recent infection for the purposes of VS case definition during an outbreak. All case definitions for VS require compatible clinical signs and have several options for diagnostic confirmation. A summary of VS case definitions used in the 2019–2020 outbreak response is presented in Table 2. An IgM capture ELISA has been developed recently at NVSL and may also be used as a reliable indicator of recent infection in future outbreaks. While the VS index case for the nation, index cases for newly affected states, and VS cases in ruminants and swine require diagnostic confirmation at NVSL, since 2015 the USDA-approved National Animal Health Laboratory Network (NAHLN) laboratories located in historically VS-affected states have been activated during outbreaks to conduct VSV testing in clinically-affected equids. This action has successfully increased laboratory capacity and reduced result turnaround time during an outbreak response.

Once an index case of VS is diagnostically confirmed in the U.S., a national situation report is issued first to state and federal animal health officials and bilateral trade partners for their awareness and then the report is publicly posted to the USDA-APHIS website [2]. At least once weekly situation reports are issued and posted throughout the outbreak thereafter until the incident is declared over, usually 30 days after the last quarantine release in the country. A joint state–federal response following standardized response protocols and using local personnel is organized in each affected state. A national-level situation unit leader is activated to provide support, maintain response continuity across states, gather data, and issue situation reports. State animal health officials provide electronic communication by mass email to private veterinarians licensed to practice in the state notifying them of the confirmation of a VS case, recommending increased surveillance and educational outreach to clients, reminding of reporting requirements, and providing instructions on response measures. Information is also posted to state animal health officials’ websites, including specifics of any new interstate movement and entry requirements enacted as a response measure.

Livestock premises with laboratory diagnostic results meeting a VS confirmed case definition are categorized as confirmed positive premises. Once a county is confirmed as VSV-positive, new equine premises presenting with clinical signs of VSV in that county are not required to be tested for confirmation of the disease, but the premises is quarantined and classified as a suspect premises. Premises are also classified as suspect if clinical animals on the premises fail to meet a confirmed case definition, but have diagnostic evidence of recent VSV infection. All confirmed positive and suspect VS premises are placed under state quarantine for a minimum of 14 days from the onset of lesions in the last affected animal on the premises. The quarantine applies to all VS-susceptible species on the premises and no movement of these species off-site is permitted without approval of the state veterinarian.

Isolation of lesioned animals from non-lesioned animals is instituted to reduce spread of the virus by direct contact and aggressive vector control recommendations are provided to be instituted by the premises/animal owner to further reduce within-herd spread. Oversight for equine premises is conducted by private veterinarians communicating with state animal health officials in most states, while oversight of ruminant and swine premises is conducted directly by state or federal animal health officials. Private veterinarians or animal health officials overseeing each premises confirm the 14-day countdown after the onset of lesions in the last affected animal. State animal health officials issue a quarantine release once this time period has passed with no new cases presenting. Continuation of aggressive vector control on the premises is recommended throughout the remainder of the outbreak, as re-infection of previously affected animals and lesion-development in new animals after quarantine release has occurred occasionally from continued presence of infected vectors in the general area when vector mitigations on the premises are inadequate.

### 2.2. 2019 and 2020 VS Outbreaks 

The 2019 VS outbreak began on 21 June 2019, when the NVSL in Ames, Iowa, confirmed the first VSV-positive (Indiana serotype) equine premises in Kinney County, Texas [2]. New Mexico, Colorado, Wyoming, Oklahoma, Nebraska, Utah, and Kansas subsequently reported cases, which were confirmed by NVSL on 26 June 2019 (Sandoval County, NM, USA), 3 July 2019 (Weld County, CO, USA), 24 July 2019 (Platte County, WY, USA), 29 July 2019 (Tillman County, OK, USA), 9 August 2019 (Lincoln County, NE, USA), 19 August 2019 (Emery and Uintah Counties, UT, USA), and 23 October 2019 (Sherman County, KS, USA).

A total of 1144 VSV-affected premises were identified in 111 counties in the eight affected states making it the largest U.S. outbreak in at least the past 40 years of recorded history. Four hundred seventy-two (472) premises were confirmed VSV-positive and 672 premises were classified as suspect premises and presumed infected. All confirmed positive cases in the 2019 outbreak were identified as VSIV serotype, which had not been isolated in the U.S. since the 1997–1998 outbreak [12]. After 1998, subsequent VS outbreaks in 2004, 2005, 2006, 2009, 2010, 2012, 2014, and 2015 had all been confirmed as VSNJV serotype [2]. One thousand one hundred twenty-eight (1128) of the 1144 total affected premises in 2019 had only equine species clinically affected, 15 premises had only affected cattle (Boulder, Delta, Garfield, Larimer, and Pitkin County, CO, USA; Gonzales County, TX, USA; Uintah County, UT, USA; and Hot Springs, Park, and Platte County, WY, USA), and one premises had both cattle and horses clinically affected (Park County, WY, USA).

The state with the most affected premises in 2019 was Colorado with 693 premises in 38 counties, followed by Texas with 172 premises in 37 counties, Wyoming with 149 premises in 11 counties, New Mexico with 76 premises in 12 counties, Utah with 26 premises in 6 counties, Nebraska with 26 premises in five counties, and Kansas and Oklahoma which each had 1 confirmed positive affected premises in one county. A listing of the number of confirmed positive and suspect premises by county in each state for the 2019 outbreak is available in Table 3. While complete inventories of susceptible animals were not available for all premises and a count of numbers of lesioned animals was only collected at the time of the initial report, there were at least 1851 lesioned equids and 32 lesioned cattle out of 9987 equids, 9009 cattle, and 1374 other susceptible species on the quarantined premises.

Reported lesion onset dates ranged from as early as 14 June 2019 to as late as 12 November 2019. Affected premises were quarantined and managed as previously described. All quarantined premises were released in Oklahoma as of 7 August 2019, New Mexico as of 5 September 2019, Texas as of 31 October 2019, Kansas as of 4 November 2019, Nebraska as of 14 November 2019, Utah as of 25 November 2019, Colorado as of 17 December 2019, and Wyoming as of 27 December 2019. Intensive surveillance and testing of any reported lesioned animals in all states continued after the final quarantine release in Wyoming; however, no further cases were confirmed and the 2019 outbreak was declared over [2].

Based on experience from previous VS outbreaks, and given the large number of infected premises and the widespread geographic distribution of the 2019 outbreak, epidemiologists and other VS experts predicted that overwintering of the virus was likely to occur and a 2020 VS outbreak was expected. As anticipated, the 2020 VS outbreak began on 13 April 2020, when the NVSL in Ames, Iowa, confirmed the first VSV-positive (Indiana serotype) equine premises in Dona Ana County, New Mexico [2]. Arizona, Texas, Kansas, Nebraska, Oklahoma, Missouri, and Arkansas subsequently reported VS cases, which were confirmed by NVSL on 22 April 2020 (Cochise County, AZ, USA), 23 April 2020 (Starr County, TX, USA), 16 June 2020 (Butler County, KS, USA), 24 June 2020 (Buffalo County, NE, USA), 7 July 2020 (Washington County, OK, USA), 13 July 2020 (Newton County, MO, USA), and 27 July 2020 (Benton County, AR, USA).

A total of 326 VSV-affected premises were identified in 70 counties in the eight affected states in 2020. Two hundred six (206) premises were confirmed VSV-positive and 120 were classified as suspect premises and presumed infected. Three hundred thirteen (313) of the 326 affected premises had only equine species clinically affected, 12 premises had clinically affected cattle (McMullen, Starr, and Zapata Counties, TX, USA; Butler, Cowley, Marion, and Montgomery Counties, KS, USA; Cedar and Ozark Counties, MO, USA), and 1 premises had both equine species and cattle clinically affected (Cedar County, MO, USA).

The state with the most affected premises in 2020 was Kansas with 196 premises in 26 counties, followed by Missouri with 54 premises in 12 counties, Oklahoma with 22 premises in nine counties, Arizona with 19 premises in seven counties, New Mexico with 16 premises in six counties, Texas with 10 premises in six counties, Nebraska with five premises in three counties, and Arkansas with four premises in one county. A listing of the number of confirmed positive and suspect premises by county in each state for the 2020 outbreak is available in Table 4. Complete inventories of susceptible animals were not available for all premises and a count of numbers of lesioned animals was only collected at the time of the initial report, but there were at least 520 lesioned equids and 24 lesioned cattle out of 2305 equids, 2557 cattle, and 222 other susceptible species on the quarantined premises.

While the majority of the confirmed VSV-positive cases in the 2020 outbreak were identified as VSIV serotype, seven premises in four counties in south and south-central Texas were confirmed with VSNJV serotype. The last time a U.S. outbreak involved both VSIV and VSNJV serotypes was in 1997–1998 [12,18].

Reported lesion onset dates ranged from as early as 6 April 2020 to as late as 29 September 2020. Affected premises identified during the 2020 outbreak were quarantined and managed as previously described. All VSV-quarantined premises were released in New Mexico as of 4 June 2020, Texas as of 8 June 2020, Arizona as of 8 July 2020, Nebraska as of 30 July 2020, Arkansas as of 19 August 2020, Oklahoma as of 26 August 2020, Kansas as of 23 September 2020, and Missouri as of 15 October 2020. Intensive surveillance and testing of any reported lesioned animals in all states continued after the final quarantine release in Missouri; however, no further cases were confirmed and the 2020 outbreak was declared over [2].

**Figure 1:**
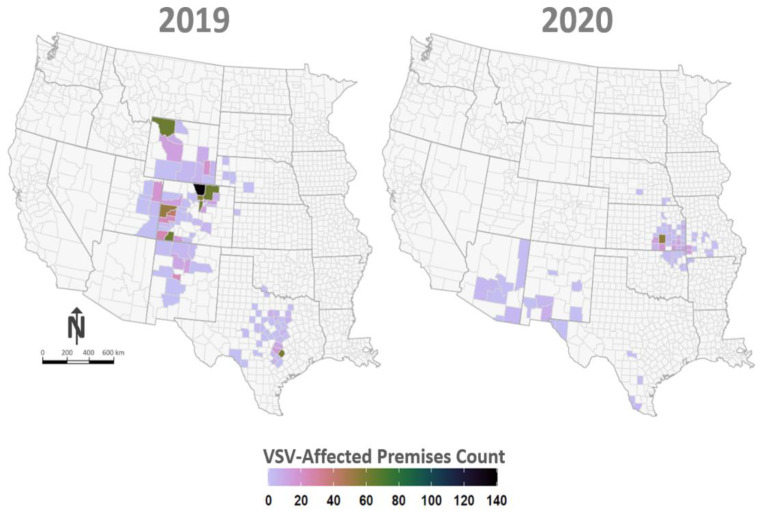
VSV-affected premises maps. Figure depicts the geographic location and number of VSV-affected premises by U.S. County. Panel at left displays VSV-affected counties documented in 2019 and panel at right shows counties recorded in 2020. Counties are color coded according to legend at bottom with darker tones (green colors) indicating relatively higher numbers of VSV-affected premises.

**Figure 2:**
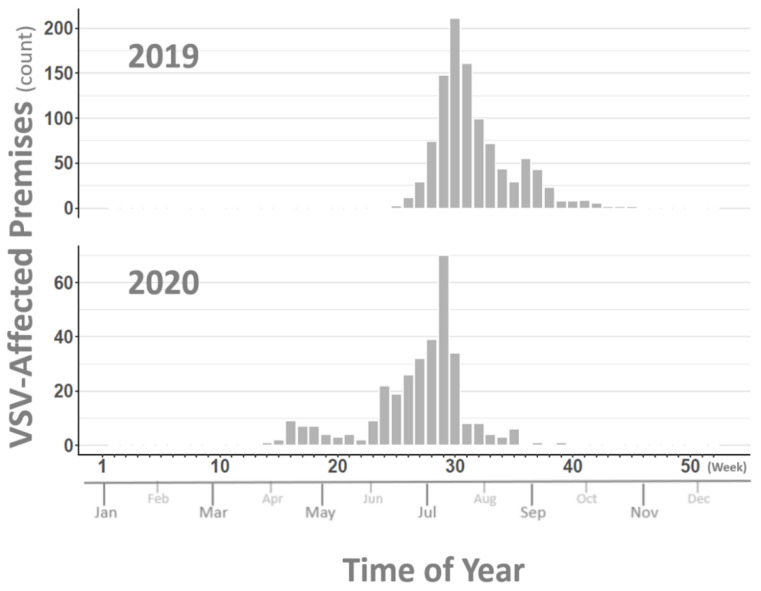
Count of VSV-affected premises by week of reported lesion onset for 2019 and 2020. Vertical axes at right correspond to bar height and describe the number of premises reporting VSV lesion onset. Major horizontal axis lists week of year (Weeks 1–52) with approximate month of year displayed along minor horizontal axis. Top panel represents premises counts for the year 2019 and bottom panel provides those for 2020.

**Figure 3:**
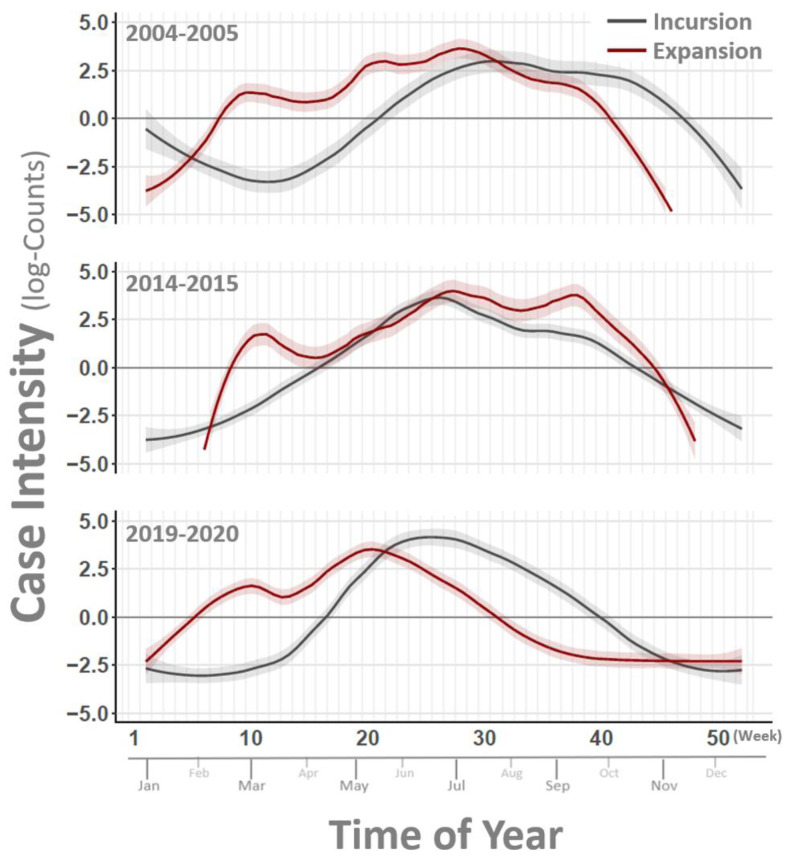
Estimated VSV case intensity. Figure compares incursion and expansion patterns for VSV outbreak cycles documented in 2004–2005 (top), 2014–2015 (center), and 2019–2020 (bottom). Vertical axes at left show case counts (logarithmic scale) and major horizontal axis lists week of year (Weeks 1–52) with approximate month of year displayed along minor horizontal axis. Curved lines represent smoothed counts of VSV-affected premises on the logarithmic scale. Shaded regions around curved lines signify the 95% Credible Interval for estimates. Dark gray line in each panel corresponds to incursion year estimates (2004, 2014, 2019) and red lines give expansion year estimates (2005, 2015, 2020). Note that outbreaks during expansion years tend to begin earlier in the year relative to incursion year outbreaks.

Widespread geographic distribution of affected premises and variability in case density between affected counties was observed in both the 2019 and 2020 outbreaks. County-level maps shaded to depict numbers of affected premises for each outbreak year are shown in Figure 1. Epidemic curves for the 2019 and 2020 outbreaks developed using lesion onset dates for each affected premises (Figure 2) are representative of case intensity rates and timelines observed in previous outbreaks, but they differ slightly from each other. The 2019 outbreak shows a single strong peak of cases from July to September, while the 2020 outbreak has a bi-modal curve with the early smaller peak being generated by cases in the southwestern states (Arizona, New Mexico, and Texas) and the second stronger peak being driven by the more eastern affected states (Arkansas, Kansas, Missouri, and Oklahoma). Additionally, the 2019 outbreak began later in the season (June) than the 2020 outbreak (April), which correlates historically to VSV behavior in an incursion year, while earlier seasonality, as seen in 2020, correlates historically to the appearance of an expansion year for VS [15]. Further support for this classification is shown by comparison of VS case intensity rates in earlier incursion/expansion year pairs, 2004–2005 and 2014–2015 (Figure 3). Case intensity rates characteristic of a VS incursion year were seen in 2004, 2014, and 2019, while those more characteristic of an expansion year were seen in 2005, 2015, and 2020. A more detailed analysis of climatic and ecological variables previously identified as indicative of VS incursion years versus expansion years is currently underway using 2019 and 2020 data.

### 2.3. 2020 Kansas VSV Vector Collection

Recent multi-year projects to evaluate potential VSV vectors in historic VS-affected areas have been ongoing in Colorado and New Mexico, but a study in Kansas was not already in progress when the VS index case in the state was confirmed on 16 June 2020. A vector trapping project was quickly developed in response to the Kansas index case and is presented here.

#### 2.3.1. Methods

**Figure 4:**
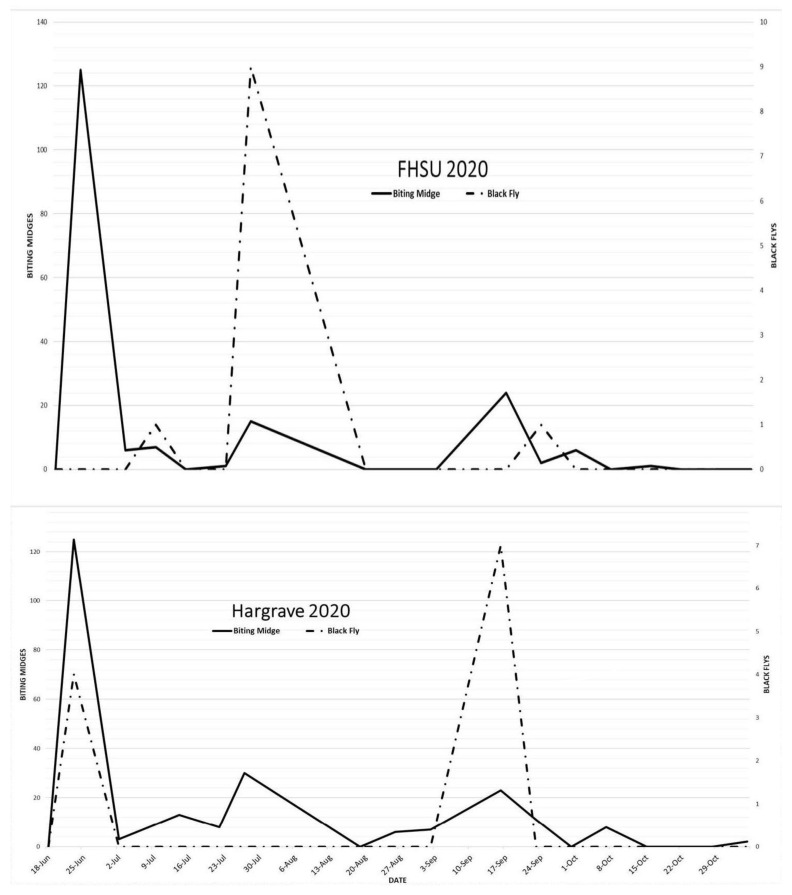
Counts of black flies and biting midges trapped at two collection sites (FHSU and Hargrave) by collection date in Kansas during the 2020 VS outbreak.

To quantify the abundance of vesicular stomatitis vector genera (Simulidae (black flies), Phlebotominae (sand flies), and Ceratopogonidae (biting midges) throughout the summer of 2020 in central Kansas, ultraviolet Centers for Disease Control insect traps with ethylene glycol preservatives were placed at cattle farms in two locations reflected in Figure 4 as “Hargrave” in Rush County and “FHSU” (Fort Hays State University) in Ellis County. Locations were selected for presence of cattle throughout the summer months, reduced wind exposure, proximity to an open water source and the absence of ambient light sources. Overnight trapping between dusk and dawn (~10 h) was conducted 18 times at each location between 18 June 2020 and 5 November 2020. Trapping was attempted once weekly, but adverse weather conditions and resource restrictions limited the ability to collect each week of the sampling period. High temperatures, long storage times, and contamination from dust and large insects can degrade insect specimens and make species identification impossible; therefore, only genera were identified at the end of the sampling period. No virus detection was attempted on the degraded samples.

#### 2.3.2. Results

Identifiable insects were collected on 15 of 18 sample collection dates with a significant overall decline in abundance after 24 September 2020. The number of biting midge detections were consistent between each location throughout the summer with the largest spike in detections occurring on 25 June 2020. Black fly detections varied temporally between the two locations with large bimodal peaks on 25 June 2020 and 17 September 2020 at the Hargrave location and a single peak on 30 July 2020 at the FHSU location. These findings suggest that both black fly and biting midge populations in central Kansas peaked prior to the VSV outbreak in Southeastern Kansas, which implies that VSV transmission may have been undetected in this area of the state. Since this part of the U.S. had not been considered a historically VS-affected region in at least the past 50 years, additional vector collection studies in the 2020 Kansas/Missouri/Oklahoma/Arkansas outbreak area are needed to further evaluate their role in VSV transmission and the potential differences in vector dynamics in this new region versus the historically VS-affected southwestern and Rocky Mountain regions.

### 2.4. 2019–2020. VSIV Preliminary Phylogenetic Analysis

Full genome sequencing and phylogenetic analysis of historic U.S.-origin VSV isolates began several years ago and is ongoing to further inform epidemiological connections between geographically distinct cases of VS and evaluate minute changes in the virus over time. Preliminary phylogenetic analysis of published full genome sequences of VSIV isolates from the 2019 and 2020 outbreak and their relationship to previously published U.S. isolates are included here.

#### 2.4.1. Methods

All publicly available full genome sequences from the U.S, 2019–2020 VSIV outbreak (*n* = 7) were downloaded from GenBank along with the available full genome sequences from field isolates collected between 1985–2001 in Central America, Mexico, and the U.S, (*n* = 10). Sequences were aligned using MUSCLE implemented in MegaX [19] and the alignment was refined to include only sequences with >95% similarity to improve resolution of relationships among the 2019–2020 sequences (Table 5). The Tamura–Nei model with uniform evolutionary rates among sites was identified as the most appropriate model, and a maximum likelihood phylogenetic tree was constructed using this model and 500 bootstrap replicates implemented in MegaX. The final consensus tree was visualized in FigTree v1.4.3 [20].

#### 2.4.2. Results

**Figure 5:**
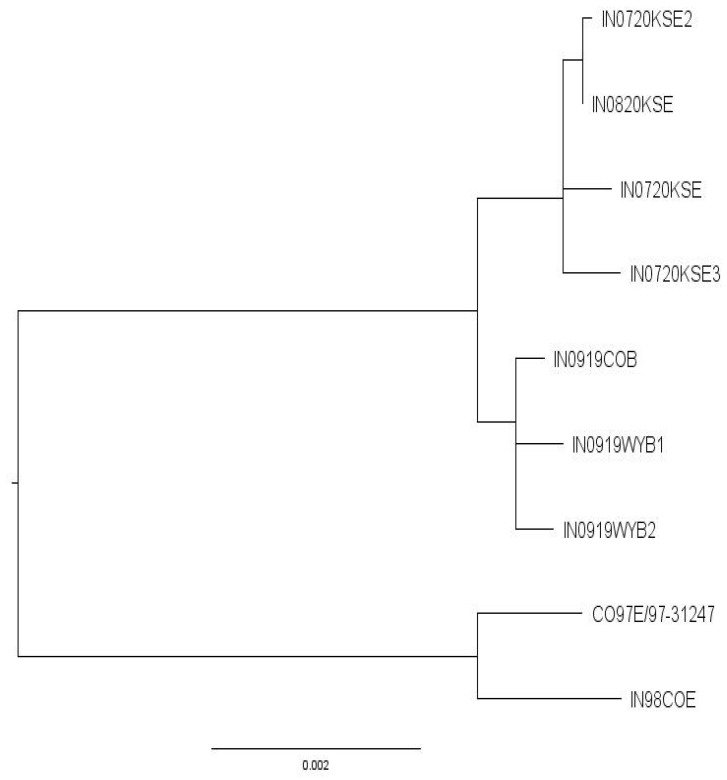
Maximum likelihood phylogenetic analysis of full-genome sequences from the 2019–2020 and 1997–1998 U.S. VSV-IN outbreaks. All nodes had >70% bootstrap support. The close relationship between the 2019 and 2020 sequences supports an overwintering event causing the 2020 outbreak.

The 2019–2020 outbreak sequences were 98.86–98.92%, similar to two isolates collected during the 1997–1998 VSIV outbreak in the U.S. No other isolate shared >95% identity across the full genome. A BLASTn search of the phosphoprotein (P) hypervariable region (450-nt) revealed additional sequences from the 1997–1998 outbreak, but no other closely related isolates. The seven 2019–2020 isolates were identical across the P hypervariable region, and 99.78–99.99% identical across the full genome. On phylogenetic analysis, the 2019–2020 outbreak formed a monophyletic clade, distinct from the 1997–1998 outbreak isolates (Figure 5). Isolates formed two distinct groups within the 2019–2020 clade; one comprised solely of 2019 isolates, and one comprised solely of 2020 isolates. The average within-group genetic identity was 99.94% for the 2019 and 2020 groups, and between-group genetic identity was 99.81%. These results strongly suggest the 2020 U.S. outbreak resulted from an overwintering virus from the 2019 outbreak, and not a separate incursion.

## 3. Discussions

The 2019 and 2020 VS outbreaks shared some characteristic features of historic outbreaks in the U.S., but also had several unexpected attributes. The factors involved that boosted the 2019 outbreak to become the largest in both size and geographic scope in the past 40 years of recorded history are still a relative mystery, although climatological and ecological conditions affecting vector abundance, dispersal, or habitat quality are suspected to be involved. Indeed, the previous round of outbreaks in 2014–2015 were also larger than normal by comparison to other years and may hold the key to identification of climate factors that may have been intensifying into 2019. Questions remain regarding what caused U.S. outbreaks to be dominated exclusively by the VSNJV serotype since the last VSIV outbreak in 1997–1998 and, subsequently, what changed that allowed VSIV to appear and surge alone so successfully in 2019. Clinically, the VSNJV and VSIV presented across the outbreaks quite similarly with the full gamut of lesion types represented and neither virus serotype looking any more or less virulent in the animals than the other.

Phylogenetic analysis suggests the occurrence of an overwintering event of VSIV between the 2019 and 2020 outbreaks. While overwintering of the virus was an expected event based on historic occurrences of the same, there were several completely unexpected outcomes that followed. Based on study of the 2004–2006 and 2014–2015 outbreaks and the dynamics previously described on incursion years versus expansion years, the 2020 outbreak was expected to begin with new cases in all the same states where last observed in 2019 and then expand outward from those saturated regions. It began as predicted with the first cases of 2020 identified early in the season and in previously affected areas in the lower southwestern states before expanding northward, apparently mirroring expected temporal peaks of vector abundance. However, the expected cases in the Rocky Mountain states (Colorado, Utah, and Wyoming) were never observed. This region with the most cases in 2019 had zero cases confirmed in 2020 despite strong surveillance and testing. We know this outcome is not due to an immunity of the previously exposed animals to the virus. High antibody titers to VSV from previous outbreak years have failed to prevent individual animals from developing lesions in the next outbreak year. Anecdotally, horse owners in historically affected VS-regions have reported that the same horse or horses in their herd developed lesions during every outbreak experienced since living there. Additionally, several animals in each outbreak are typically identified presenting with new lesions after the previous lesions have healed on premises where the vector control is determined to have been inadequate. These cases suggest no resistance to infected vector re-exposure with the same virus, despite very high antibody titers, and necessitate the premises be re-quarantined and a more aggressive vector control program administered. There were five such cases documented during the 2019–2020 outbreak.

One hypothesis for the 2020 absence of VS cases in the Rocky Mountain region is that the environmental conditions in the area did not support the high-volume of black flies and *Culicoides* spp. that were present in 2019. Specifically, Colorado, Utah, and Wyoming were experiencing extreme drought conditions throughout 2020, which may have impacted the vector hatch and overall insect populations. Further in-depth study is planned to evaluate this hypothesis and investigate other potential causes.

Another unexpected outcome in the 2020 outbreak was the development of a new outbreak region in the Kansas/Missouri/Oklahoma/Arkansas area. While Kansas and Oklahoma each confirmed a single VSV-infected premises in 2019 in counties bordering active VSV-infected states, neither state had previously reported cases in at least the past 50 years. Kansas and Oklahoma were anticipated to identify more cases in 2020 in western portions of the states where 2019 cases were found, but instead, the 2020 outbreak erupted far to the east in both states and spilled over into western Missouri and northwest Arkansas. We presented vector collection data here indicating that VSV-infection in central Kansas may have pre-dated the southeastern Kansas cases and been missed, but more study is needed to evaluate how the virus moved and flourished further east than expected. Full genomic sequencing and phylogenetic analysis of the viral isolates from this region are planned to determine the geographic distribution of other closely related lineages from 2020, which might indicate the source of a progenitor virus.

Finally, the new 2020 incursion of a VSNJV serotype virus in south Texas was another unexpected development during the outbreak. Texas, in fact, had cases of both VSIV and VSNJV simultaneously in 2020. While the new VSNJV incursion began in Starr and Zapata Counties along the Mexican border in deep south Texas and moved directly northward to McMullen and Kerr Counties in south-central Texas, cases of VSIV were simultaneously confirmed in far west Texas in El Paso and Hudspeth Counties. While it stands to reason that west Texas was geographically involved in the same VSIV outbreak as its neighboring state New Mexico, what is more perplexing was the finding of both a VSIV-infected premises and a VSNJV-infected premises with lesion onset dates within 2 days of each other and located approximately 28 miles apart in Zapata County, Texas. No other VSIV affected premises were found in south Texas and the nearest VSIV infected premises in west Texas was approximately 450 miles away. It is unknown what if any VS cases were occurring on the other side of the border in Mexico at the same time, which could better explain the situation. Full genomic sequencing and phylogenetic analysis are planned to investigate the potential origin of both viruses and the relationship of the 2019 and 2020 isolates to viruses circulating more recently in Mexico.

## Figures and Tables

**Table 1:** Summary of VS outbreaks in the U.S. within the past 20 years including outbreak year, affected states, virus serotype, and number of affected livestock premises.

Outbreak Year	Number States Affected	States	VSV Serotype	Number Affected Premises
2004	3	CO, NM, TX	VSNJV	294
2005	9	AZ, CO, ID, MT, NE, NM, TX, UT, WY	VSNJV	445
2006	1	WY	VSNJV	13
2009	2	NM, TX	VSNJV	5
2010	1	AZ	VSNJV	2
2012	2	CO, NM	VSNJV	36
2014	4	AZ, CO, NE, TX	VSNJV	435
2015	8	AZ, CO, NE, NM, SD, TX, UT, WY	VSNJV	823
2019	8	CO, KS, NE, NM, OK, TX, UT, WY	VSIV	1144
2020	8	AR, AZ, KS, MO, NE, NM, OK, TX	VSIV, VSNJV (TX)	326

State abbreviations: AR-Arkansas, AZ-Arizona, CO-Colorado, ID-Idaho, KS-Kansas, MO-Missouri, MT-Montana, NE-Nebraska, NM-New Mexico, OK-Oklahoma, SD-South Dakota, TX-Texas, UT-Utah, WY-Wyoming.

**Table 2:** Case definitions used in the 2019 and 2020 VS outbreaks.

Case Classification	Case Definition
Index Case for the Nation	Compatible clinical signs of VS and laboratory confirmation at NVSL including one or more of the following: Virus isolation of VSV Viral genome sequence data indicative of VSV Four-fold change in complement fixation test (CFT) titer in paired sera collected at least 7 days apart Four-fold increase in virus neutralization (VN) titer in paired sera collected at least 7 days apart
Index Case for Subsequent States (after Index Case for the Nation is met)	Compatible clinical signs of VS and laboratory confirmation at NVSL including one or more of the following: Virus isolation of VSV Viral genome sequence data indicative of VSV Four-fold change in CFT titer in paired sera collected at least 7 days apart Four-fold increase in VN titer in paired sera collected at least 7 days apart Positive CFT titer > 1:40
Subsequent Confirmed Case in a VSV-positive State	Compatible clinical signs of VS and laboratory confirmation at either NVSL or a VS-activated NAHLN laboratory including one or more of the following: Virus isolation of VSV Viral genome sequence data indicative of VSV Real-time RT-PCR detection of VSV Four-fold increase in VN titer in paired sera collected at least 7 days apart Positive CFT titer > 1:5
Suspect Case in a VSV-positive State	A case may be classified as “suspect” in the following situations: An equid with compatible clinical signs of VS, without diagnostic confirmation, but located in a confirmed VSV-positive county A susceptible species of livestock with compatible clinical signs of VS that does not meet one of the confirmed case definitions above, but has diagnostic evidence of recent VSV infection

**Table 3:** Location, classification, and count of VSV-affected premises in 2019.

	Confirmed Positive Premises	Suspect Premises	Total Premises Quarantined
COLORADO			
Adams County	7	8	15
Alamosa County	2	2	4
Arapahoe County	2	0	2
Archuleta County	7	10	17
Boulder County	24	35	59
Broomfield County	1	1	2
Chaffee County	3	0	3
Clear Creek County	1	0	1
Conejos County	2	0	2
Delta County	5	40	45
Dolores County	1	0	1
Douglas County	14	3	17
Eagle County	2	0	2
El Paso County	1	0	1
Fremont County	2	2	4
Garfield County	17	3	20
Gilpin County	1	0	1
Grand County	1	0	1
Gunnison County	2	0	2
Jefferson County	36	28	64
La Plata County	10	56	66
Larimer County	57	87	144
Las Animas County	1	0	1
Mesa County	12	47	59
Mineral County	1	0	1
Moffat County	1	0	1
Montezuma County	3	26	29
Montrose County	4	25	29
Morgan County	2	5	7
Ouray County	3	2	5
Park County	2	0	2
Pitkin County	3	0	3
Pueblo County	2	3	5
Rio Blanco County	2	1	3
San Miguel County	1	4	5
Summit County	1	1	2
Teller County	1	0	1
Weld County	38	29	67
TOTAL: 38 COUNTIES	275	418	693
KANSAS			
Sherman County	1	0	1
TOTAL: 1 COUNTY	1	0	1
NEBRASKA			
Dawes County	1	0	1
Garden County	1	0	1
Lincoln County	1	0	1
Morrill County	2	1	3
Scotts Bluff County	11	9	20
TOTAL: 5 COUNTIES	16	10	26
NEW MEXICO			
Cibola County	1	0	1
Los Alamos County	1	0	1
Mora County	1	0	1
Rio Arriba County	1	6	7
Sandoval County	7	3	10
San Juan County	1	3	4
San Miguel County	2	2	4
Santa Fe County	5	6	11
Sierra County	1	0	1
Socorro County	1	0	1
Taos County	5	1	6
Valencia County	21	8	29
TOTAL: 12 COUNTIES	47	29	76
OKLAHOMA			
Tillman County	1	0	1
TOTAL: 1 COUNTY	1	0	1
TEXAS			
Bastrop County	9	50	59
Bell County	1	2	3
Bosque County	1	0	1
Brown County	1	0	1
Caldwell County	2	1	3
Coleman County	3	0	3
Collin County	3	0	3
Coryell County	1	0	1
Dallas County	4	0	4
Eastland County	1	0	1
Ellis County	4	2	6
Erath County	1	1	2
Falls County	1	0	1
Gonzales County	1	0	1
Guadalupe County	1	5	6
Haskell County	1	0	1
Hays County	1	4	5
Hill County	1	0	1
Hood County	4	3	7
Johnson County	1	0	1
Kerr County	1	0	1
Kinney County	1	0	1
Lampasas County	1	0	1
Mason County	1	0	1
McLennan County	2	3	5
Mills County	1	0	1
Palo Pinto County	6	1	7
Parker County	4	1	5
San Saba County	1	0	1
Shackelford County	2	0	2
Somervell County	2	1	3
Taylor County	1	0	1
Tom Green County	3	0	3
Travis County	2	15	17
Val Verde County	1	0	1
Wichita County	1	0	1
Williamson County	4	7	11
TOTAL: 37 COUNTIES	76	96	172
UTAH			
Carbon County	1	0	1
Duchesne County	1	0	1
Emery County	1	0	1
Grand County	2	1	3
San Juan County	1	0	1
Uintah County	7	12	19
TOTAL: 6 COUNTIES	13	13	26
WYOMING			
Albany County	5	1	6
Big Horn County	1	1	2
Carbon County	3	2	5
Converse County	3	6	9
Fremont County	8	5	13
Goshen County	1	5	6
Hot Springs County	5	11	16
Laramie County	1	0	1
Park County	10	61	71
Platte County	5	14	19
Sweetwater County	1	0	1
TOTAL: 11 COUNTIES	43	106	149
TOTAL PREMISES ALL STATES	472	672	1144

**Table 4:** Location, classification, and count of VSV-affected premises in 2020.

	Confirmed Positive Premises	Suspect Premises	Total Premises Quarantined
ARIZONA			
Apache County	2	0	2
Cochise County	4	0	4
Gila County	1	0	1
Maricopa County	7	1	8
Pima County	1	0	1
Pinal County	2	0	2
Santa Cruz County	1	0	1
TOTAL: 7 COUNTIES	18	1	19
ARKANSAS			
Benton County	4	0	4
TOTAL: 1 COUNTY	4	0	4
KANSAS			
Allen County	3	5	8
Bourbon County	1	0	1
Butler County	31	24	55
Chase County	1	1	2
Cherokee County	6	7	13
Coffey County	1	2	3
Cowley County	9	8	17
Crawford County	1	1	2
Elk County	1	0	1
Franklin County	1	0	1
Greenwood County	2	2	4
Harvey County	1	0	1
Johnson County	1	1	2
Labette County	2	4	6
Linn County	2	1	3
Lyon County	3	2	5
Marion County	2	0	2
Miami County	3	6	9
Montgomery County	6	10	16
Morris County	1	1	2
Neosho County	3	10	13
Riley County	1	0	1
Sedgwick County	13	1	14
Sumner County	2	4	6
Wilson County	3	5	8
Woodson County	1	0	1
TOTAL: 26 COUNTIES	101	95	196
MISSOURI			
Camden County	2	0	2
Cedar County	3	0	3
Dallas County	2	0	2
Douglas County	1	0	1
Jasper County	8	6	14
Lawrence County	1	1	2
McDonald County	5	2	7
Newton County	8	7	15
Ozark County	3	1	4
Phelps County	2	0	2
St. Clair County	1	0	1
Texas County	1	0	1
TOTAL: 12 COUNTIES	37	17	54
NEBRASKA			
Buffalo County	3	0	3
Gage County	1	0	1
Johnson County	1	0	1
TOTAL: 3 COUNTIES	5	0	5
NEW MEXICO			
Bernalillo County	1	0	1
De Baca County	2	0	2
Dona Ana County	6	1	7
Eddy County	1	0	1
Grant County	1	0	1
Sierra County	2	2	4
TOTAL: 6 COUNTIES	13	3	16
OKLAHOMA			
Adair County	1	0	1
Cherokee County	3	0	3
Craig County	1	1	2
Nowata County	1	0	1
Osage County	2	1	3
Ottawa County	4	0	4
Rogers County	1	2	3
Tulsa County	1	0	1
Washington County	4	0	4
TOTAL: 9 COUNTIES	18	4	22
TEXAS			
El Paso County	1	0	1
Hudspeth County	1	0	1
Kerr County	1	0	1
McMullen County	1	0	1
Starr County	4	0	4
Zapata County	2	0	2
TOTAL: 6 COUNTIES	10	0	10
TOTAL PREMISES ALL STATES	206	120	326

**Table 5:** GenBank accession number and metadata for sequences included in the phylogenetic analyses.

Sequence Name	Location	Collection Date	GenBank Accession
IN0919COB	Colorado	18 Sep 2019	MT437285
IN0919WYB1	Wyoming	5 Sep 2019	MT437284
IN0919WYB2	Wyoming	9 Sep 2019	MT437283
IN0720KSE	Kansas	Jul 2020	MW373776
IN0720KSE2	Kansas	Jul 2020	MW373777
IN0720KSE3	Kansas	Jul 2020	MW373778
IN0820KSE	Kansas	Aug 2020	MW373779
IN98COE	Colorado	1998	AF473864
CO97E/97-31247	Colorado	Aug 1997	MK934319

## Data Availability

Summary data from VS cases are publicly available from the USDA-APHIS website at the following link: https://www.aphis.usda.gov/aphis/ourfocus/animalhealth/animal-disease-information/cattle-disease-information/vesicular-stomatitis-info (accessed on 11 June 2021). Full genome sequencing data analyzed in this study is publicly available from GenBank: https://www.ncbi.nlm.nih.gov/genbank/ (accessed on 11 June 2021).

## References

[1] Rodríguez L.L. (2002). Emergence and Re-Emergence of Vesicular Stomatitis in the United States. Virus Res..

[2] Vesicular Stomatitis Outbreak Situation Reports on USDA-APHIS. https://www.aphis.usda.gov/aphis/ourfocus/animalhealth/animal-disease-information/cattle-disease-information/vesicular-stomatitis-info.

[3] Pelzel-McCluskey A.M., Winter A.L. (2020). Vesicular Stomatitis. Merck Veterinary Manual.

[4] Duarte P.C., Morley P.S., Traub-Dargatz J.L., Creekmore L.H. (2008). Factors Associated with Vesicular Stomatitis in Animals in the Western United States. J. Am. Vet. Med. Assoc..

[5] Mohler J.R. (1918). Vesicular Stomatitis of Horses and Cattle.

[6] Hanson R.P. (1952). The natural history of vesicular stomatitis. Bacteriol. Rev..

[7] Schmidtmann E.T., Tabachnick W.J., Hunt G.J., Thompson L.H., Hurd H.S. (1999). 1995 Epizootic of Vesicular Stomatitis (New Jersey Serotype) in the Western United States: An Entomologic Perspective. J. Med. Entomol..

[8] Tesh R.B., Boshell S.J., Modi G.B., Morales A.A., Young D.G., Corredor A.A., De Carrasquilla C.F., De Rodriguez C., Walters L.L., Gaitan M.O. (1987). Natural Infection of Humans, Animals, and Phlebotomine Sand Flies with the Alagoas Serotype of Vesicular Stomatitis Virus in Colombia. Am. J. Trop. Med. Hyg..

[9] Schnitzlein W., Reichmann M. (1985). Characterization of New Jersey Vesicular Stomatitis Virus Isolates from Horses and Black Flies during the 1982 Outbreak in Colorado. Virology.

[10] Elias E., McVey D.S., Peters D., Derner J., Pelzel-McCluskey A., Schrader T.S., Rodriguez L. (2018). Contributions of Hydrology to Vesicular Stomatitis Virus Emergence in the Western USA. Ecosystems.

[11] Rainwater-Lovett K., Pauszek S.J., Kelley W.N., Rodriguez L.L. (2007). Molecular Epidemiology of Vesicular Stomatitis New Jersey Virus from the 2004–2005 US Outbreak Indicates a Common Origin with Mexican Strains. J. Gen. Virol..

[12] Rodriguez L.L., Bunch T.A., Fraire M., Llewellyn Z.N. (2000). Re-Emergence of Vesicular Stomatitis in the Western United States Is Associated with Distinct Viral Genetic Lineages. Virology.

[13] Velazquez-Salinas L., Pauszek S.J., Zarate S., Basurto-Alcantara F.J., Verdugo-Rodríguez A., Perez A.M., Rodriguez L.L. (2014). Phylogeographic Characteristics of Vesicular Stomatitis New Jersey Viruses Circulating in Mexico from 2005 to 2011 and Their Relationship to Epidemics in the United States. Virology.

[14] Mason J., Herrera Saldaña A., Turner W.J. Vesicular Stomatitis in Mexico. Proceedings of the Annual Meeting of the United States Animal Health Association.

[15] Peters D.P.C., McVey D.S., Elias E.H., Pelzel-McCluskey A.M., Derner J.D., Burruss N.D., Schrader T.S., Yao J., Pauszek S.J., Lombard J. (2020). Big Data-Model Integration and AI for Vector-Borne Disease Prediction. Ecosphere.

[16] Pelzel-McCluskey A.M. (2015). Economic impacts of vesicular stomatitis outbreaks. Equine Dis. Q..

[17] Toms D., Powell M., Redlinger M., Beach T., Jenkins-Moore M., Buffington T., Harding C., Swenson S. Monitoring of Four Naturally Infected Horses for Vesicular Stomatitis Antibody. Proceedings of the Annual Meeting American Association of Veterinary Laboratory Diagnosticians.

[18] McCluskey B.J., Hurd H.S., Mumford E. (1999). Review of the 1997 Outbreak of Vesicular Stomatitis in the Western United States. J. Am. Vet. Med. Assoc..

[19] Kumar S., Stecher G., Li M., Knyaz C., Tamura K., Battistuzzi F.U. (2018). MEGA X: Molecular Evolutionary Genetics Analysis across Computing Platforms. Mol. Biol. Evol..

[20] Rambaut A. (2006–2018). Fig Tree. Tree Figure Drawing Tool. http://tree.bio.ed.ac.uk/software/figtree.

